# Net hepatic release of glucose from precursor supply in ruminants: a meta-analysis

**DOI:** 10.1017/S1751731119003410

**Published:** 2020-07

**Authors:** C. Loncke, P. Nozière, J. Vernet, H. Lapierre, L. Bahloul, M. Al-Jammas, D. Sauvant, I. Ortigues-Marty

**Affiliations:** 1 INRA, UMR1213 Herbivores, Saint-Genès-Champanelle F-63122, France; 2 Clermont Université, VetAgro Sup, UMR1213 Herbivores, BP 10448, Clermont-Ferrand F-63000, France; 3 Sherbrooke Research and Development Centre, Agriculture and Agri-Food Canada, QC J1M 0C8, Canada; 4 UMR Modélisation Systémique Appliquée aux Ruminants, INRA, AgroParisTech, Université Paris-Saclay, Paris 75005, France

**Keywords:** nutrient flux, gluconeogenesis, meta-analysis, glucose, liver

## Abstract

For their glucose supply, ruminants are highly dependent on the endogenous synthesis in the liver, but despite the numerous studies that evaluated hepatic glucose production, very few simultaneously measured hepatic glucose production and uptake of all precursors. As a result, the variability of precursor conversion into glucose in the liver is not known. The present study aimed at investigating by meta-analysis the relationships between hepatic glucose net release and uptake of precursors. We used the FLuxes of nutrients across Organs and tissues in Ruminant Animals database, which gathers international results on net nutrient fluxes at splanchnic level measured in catheterized animals. Response equations were developed for intakes up to 41 g DM intake/kg BW per day of diets varying from 0 to 100 g of concentrate/100 g DM in the absence of additives. The net hepatic uptake of propionate, *α*-amino-N and l-lactate was linearly and better related to their net portal appearance (**NPA**) than to their afferent hepatic flux. Blood flow data were corrected for lack of deacetylation of the para-aminohippuric acid, and this correction was shown to impact the response equations. To develop response equations between the availability of precursors (portal appearance and hepatic uptake) and net glucose hepatic release, missing data on precursor fluxes were predicted from dietary characteristics using previously developed response equations. Net hepatic release of glucose was curvilinearly related to hepatic supply and uptake of the sum of precursors, suggesting a lower conversion rate of precursors at high precursor supply. Factors of variation were explored for the linear portion of this relationship, which applied to NPA of precursors ranging from 0.99 to 9.60 mmol C/kg BW per h. Hepatic release of glucose was shown to be reduced by the portal absorption of glucose from diets containing bypass starch and to be increased by an increased uptake of *β*-hydroxybutyrate indicative of higher body tissue mobilization. These relationships were affected by the physiological status of the animals. In conclusion, we established equations that quantify the net release of glucose by the liver from the net availability of precursors. They provide a quantitative overview of factors regulating hepatic glucose synthesis in ruminants. These equations can be linked with the predictions of portal absorption of nutrients from intake and dietary characteristics, and provide indications of glucose synthesis from dietary characteristics.

## Implications

Glucose, a key nutrient for ruminants, is synthesized in the liver from a range of precursors. The response equations developed from meta-analysis highlight the variable rate of conversion of precursors into glucose. This rate follows a law of diminishing return at high precursor availability and increases with peripheral glucose demand or negative energy balance. Also glucose supplied from bypass starch partially substitutes for glucose synthesized in the liver. These results can be used in precision feeding strategies aiming at close coverage of glucose requirements. They will also contribute to the development of nutrient-based feeding systems for ruminants.

## Introduction

Glucose is a key energy yielding nutrient for all mammals. It is essential for brain, foetal growth, gut and muscle metabolism (Hammon *et al.*, 2016) and is the major precursor of lactose, the milk sugar. The ruminants have this peculiarity that they constantly need to synthesize glucose, because limited amounts, if any, are absorbed into the portal circulation unless high-starch diets are fed (Reynolds, 2006; Loncke *et al.*, 2009c). Glucose is synthesized in the liver and the kidneys, and the liver is the most important glucose synthesizing organ in ruminants (Brockman, 2005). The availability of glucose precursors is critical to support glucose synthesis.

Despite the numerous studies that evaluated net hepatic glucose release, a limited number of studies simultaneously measured the whole range of precursor uptake and conversion into glucose. Liver uptake of glucogenic precursors accounted for the majority of C released as glucose in maintenance fed sheep (e.g. El-Sabagh *et al.*, 2013) but failed to do so in *postpartum* dairy cows as reviewed by Larsen and Kristensen (2013), even after accounting for methodological effects on liver blood flow measurements. We postulated that a meta-analysis on published data would provide an overview of hepatic conversion of glucogenic precursors into glucose across a wide range of feeding and production conditions in ruminants, as in Loncke *et al.* (2015) for ketogenic nutrients across the liver.

The objectives of the present work were to investigate published knowledge on hepatic nutrient fluxes in both sheep and cattle in different physiological status and explore the quantitative relationships between the availability of major precursors, that is, propionate, amino acids and l **-**lactate, and the net hepatic release of glucose, as well as the regulatory factors which could impact these relationships. We first tested whether net hepatic fluxes of precursors responded to changes in total afferent flux (flux of nutrient supplied to the liver via the portal vein and the hepatic artery, see Supplementary Material S1) to the liver or to net portal appearance (**NPA**). Having demonstrated better relationships with NPA, the study focused on the incremental responses of hepatic fluxes to changes in NPA, thereby describing the responses to increments of ‘net’ supply of nutrients as in Loncke *et al.* (2015). The impact of analytical methods used to determine liver blood flow was tested. Factors driving the net hepatic uptake of insulin and interrelationships with glucose fluxes were also investigated.

## Material and methods

### Selection of relevant publications from the FLuxes of nutrients across Organs and tissues in Ruminant Animals database

We used the Flora (FLuxes of nutrients across Organs and tissues in Ruminant Animals; Vernet and Ortigues-Marty, 2006) database built from approximately 250 international publications on splanchnic fluxes in multi-catheterized ruminants. Publications anterior to 2008 were used for model development. To be eligible, publications had to report the net hepatic fluxes (see Supplementary Material S1 for definitions) of glucose or one of its major precursors, propionate, total amino acids (measured in the selected publication by its proxy *α*-amino-N) and l-lactate (Hammon *et al.*, 2016). Publications or treatments reporting intra-venous infusion of nutrients, use of digestion or metabolism modifiers or non-steady state feeding were discarded. Five eligible datasets were identified to study propionate, *α*-amino-N, l-lactate, glucose and infusion of propionate with respectively 29 (69), 26 (65), 25 (66), 46 (106) and 5 (16) experimental groups (treatments). No publication was available that reported total amino acids fluxes, only *α*-amino-N fluxes were available. Insulin concentrations and fluxes were also considered in a separate dataset when available (9 experimental groups, 19 treatments). The list of references used for the meta-analysis is given in Supplementary Material S2.

Because the selected publications did not systematically report the chemical composition and nutritional value of feeds and diets, the feeds and diets from all relevant publications were described according to the INRA Feed Evaluation System (2007) as detailed by Loncke *et al.* (2009c and 2015). As the nutritional status of the animals differed among publications and treatments, the availability of endogenous precursors (alanine and glycerol) coming from tissue mobilization (Leng, 1970) was estimated, when relevant, from calculated energy balance (Loncke *et al.*, 2015) and tissue composition of mobilized energy (Supplementary Material Table S1). Because in all these experiments animals were fed with frequent meal distributions to keep nutrient supply and animal metabolism at steady state, it was considered that glycogen stores remained constant and did not contribute to net hepatic glucose output.

Attention was paid to the analytical methods used to determine net hepatic fluxes to ensure that all results could be combined in the meta-analysis, as described in Supplementary Material S3 and Supplementary Material Table S2. A special focus was made on blood or plasma flow measurements, usually based on the para-aminohippuric acid (**pAH**) down-stream dilution method. The pAH is acetylated across the liver and therefore incompletely recovered in the hepatic veins if not deacetylated, as demonstrated in sheep by Katz and Bergman (1969) and in cows by Kristensen *et al.* (2009) and Rodriguez-Lopez *et al.* (2014) but 80.4% of available publications did not correct for it.

To relate net glucose flux in the same unit as its precursors, the fluxes were expressed in C units when glucose and precursors were included in the same model (3 mol C/mol propionate, l-lactate or glycerol, 6 mol C/mol glucose). The C supplied from total amino acids was calculated from the average C content of total amino acids released in the portal blood and taken up by the liver (assumed to be 4 mol C/mol amino acid-N, based on Reynolds *et al.* (1988)) and the fluxes of total amino acids. For this, fluxes of *α*-amino-N were converted to total amino acids (mmol/kg BW per h) as 1.3958 × moles of *α*-amino-N (Martineau *et al.*, 2009). All absorbed amino acids were assumed to be glucogenic, no correction was applied for the non-glucogenic leucine and lysine which represented only *circa* 6% of net amino acid C uptake by the liver (Blouin *et al.*, 2002; Doepel *et al.*, 2009) that we considered as negligible. The alanine mobilized from muscle was also considered as glucogenic based on the evidence reviewed by Larsen and Kristensen (2013) that alanine is the only amino acid involved in the inter-organ transfer of nitrogen in *postpartum* dairy cows. Subsequently and for the sake of accuracy, reference is made to *α*-amino-N for all results derived from measured and reported *α*-amino-N fluxes and expressed in moles of *α*-amino-N, and reference is made to total amino acids for all results converted into moles of C from total amino acids from *α*-amino-N.

### Meta-analyses

The meta-analyses focused on the responses of the different net hepatic fluxes (*Y* variables) to variations in explanatory variables (*X*) intra-studies, as detailed in Sauvant *et al.* (2008) and Loncke *et al.* (2015). Net hepatic fluxes are defined as the difference between hepatic efferent and afferent fluxes of nutrients measured in blood or plasma, as detailed in Loncke *et al.* (2015). Net fluxes can be either positive indicating a net hepatic release or negative indicating a net uptake. Net portal appearance is calculated from the difference between portal venous and arterial fluxes.

#### Description of the meta-design

The meta-design was described by statistics (mean, SD, range of values) generated for each parameter in the selected datasets. Species and physiological status effects were tested by one-way ANOVA. Normal data distribution and homogeneity of variances were tested by Shapiro–Wilk and Levene tests. All data (nutrient intake, NPA and net hepatic fluxes) were expressed as a function of BW to ensure normal distribution of the variables when combined across species (Loncke *et al.*, 2009c).

#### Models of net hepatic flux of nutrients

For each glucogenic precursor (propionate, total amino acids, l-lactate) it was initially tested whether their net hepatic uptake was best related to their total afferent flux or their NPA. Because net hepatic uptake was better related to net portal fluxes than to afferent fluxes, models developed included the net portal fluxes only. For l-lactate, the energy balance of the animals was also considered as a predictor because of the role of l-lactate in the C economy in the body. Second, the potential contribution of each precursor to net hepatic glucose release was evaluated by studying the relationships between the net hepatic glucose release and the availability of each precursor considered individually. Third, the net hepatic glucose release was considered in relation to the sum of all precursors. This last step required that all precursor and glucose fluxes had been measured in any given study. This was not always the case. An all-precursor dataset was constituted with studies that reported the net hepatic release of glucose and the NPA of at least one precursor (*n*_experimental groups_ = 45; *n*_treatments_ = 104). When missing, NPA of other precursors was estimated according to Loncke *et al.* (2009c), and their net hepatic uptakes were estimated according to the relationships developed in this work. Estimations represented 41%, 29% and 40% of NPA data and 43%, 28% and 46% of net hepatic flux data for propionate, l-lactate and *α*-amino-N, respectively. Estimations were evaluated by comparing predicted and observed values when available. No biases were detected (results not shown).

#### Data coding

Experimental groups were coded to specifically explore intra-study variations in NPA or net hepatic uptake of nutrients (*X* predictors). The physiological status was also coded for each study because of its potential influence on nutrient net hepatic flux (Loncke *et al.*, 2015). Within experiments, data were considered only if the reported *X* variable varied by more than 2% within-study, as in Loncke *et al.* (2015). This conservative threshold eliminated studies with absolutely no variation in *X* variables but retained studies with small variations. No selection was imposed on the *Y* variables.

#### Determination of response equations

Within-study relationships between *Y* (net hepatic flux of nutrient) and the potential explanatory variable *X* were studied using a variance–covariance GLM model:

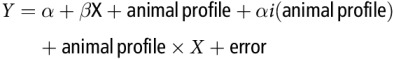

where *α* is the overall intercept and *αi* is the effect of study *i* on intercept *α*. The study effect is nested within animal profile, that is, animal species or physiological state. Parameter *β* is the slope of the overall relationship. When relevant for hepatic glucose release, non-linear models were also considered:

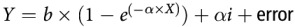

where *α* is the overall intercept and *αi* is the effect of study *i* on intercept *α*.

In all cases, the best fit model (based on the minimization of the RMSE and the SE associated with the parameters, the maximization of the adjusted *R*^2^ and the less number of interfering factors) was selected. Studies were considered as fixed factors in the models because experimental conditions and methods are specific to each study and could influence the study effect. This strategy has no impact on the statistical effect of the covariate (Sauvant *et al.*, 2008). The range of inference for this meta-analysis is, therefore, limited to the domain of the specific experiments in the dataset (Sauvant *et al.*, 2008). When the study effect was not considered, it was specified in the results. All statistical analyses were carried out using Minitab (Version 17). The normality of residuals was checked and outliers were identified on the basis of residues, HI leverage, Cook’s distance and DFITS, as in Loncke *et al.* (2009c and 2015).

#### Determination of factors influencing the response equations

To explore the causes of heterogeneity between studies, all the factors which potentially altered the relationships between *X* and *Y* variables were considered and tested as interfering factors on the model parameters (residuals, individual within-study slopes and least-squares means (**LSMeans**)) as in Loncke *et al.* (2015). This recognizes the non-random character of some of the variability present in the data and evaluates a ‘true’ within-study response. The impact of qualitative factors (analytical methods, blood or plasma concentration, physiological state and animal species) was tested by one way ANOVA. The impact of quantitative factors (nutrient and hormonal concentrations and fluxes as available, intake level and diet composition) was established by regression between model parameters and potential quantitative interfering factors. Only the significant interfering factors are reported.

#### Optimization of the response equations

When an interfering factor had been detected as significant and if not correlated to the main explanatory variable *X*, the benefits of adding it as another *X* variable to the model were evaluated on the fit (Sauvant *et al.*, 2008; Loncke *et al.*, 2015). If improvement was not significant, the interfering factor was excluded from the final response equation. The model was considered stable when the optimization analyses showed no more significant interfering factors on slopes, residuals and LSMeans or no significant improvement of fit. Parameters and correlations were considered significant at *P* ≤ 0.05, whereas 0.05 < *P* ≤ 0.10 indicated a trend. The RMSE was considered low when lower than 15% of the mean value of the predicted variable. This contributed to the good fit of the model.

## Results

### Description of the meta-design

Data were normally distributed and variances were homogenous in the different datasets. Differences in intake, dietary composition and fluxes of nutrients were greater between physiological status (non-productive adults, growing, gestating or lactating animals; Table 1) than between species (bovine or ovine; *P* < 0.001). As a result, the physiological status was included in the models, and interpretation accounted for the confounding effects of diet intake and composition, and animal species. Differences in intake and dietary composition and fluxes of nutrients according to species are however detailed in Supplementary Material S4 and Supplementary Material Tables S3a and b.

**Table 1:** Description of animals (non-productive, growing, gestating or lactating ruminants) and diets used for the meta-analysis^a^

	Non-productive adults^b^					Growing animals					Gestating animals					Lactation^c^					Physiological status effect *P*-value
	*n*_t_ sheep = 81; *n*_t_ cattle = 7					*n*_t_ sheep = 7; *n*_t_ cattle = 26					*n*_t_ sheep = 5; *n*_t_ cattle = 8					*n*_t_ sheep = 2; *n*_t_ cattle = 21					
	*n*_t_	Mean	SD	Min	Max	*n*_t_	Mean	SD	Min	Max	*n*_t_	Mean	SD	Min	Max	*n*_t_	Mean	SD	Min	Max	
Dietary composition^d^ (g/kg DM)																					
Crude fibre	88	258	92.5	39.5	404	33	194	94.6	87.9	371	13	259	62.2	177	374	23	175	57.9	107	280	<0.001
NDF	88	500	156	133	712	33	382	163	181	710	13	481	76.2	352	564	23	372	91.6	210	506	<0.001
ADF	88	279	96	43.8	393	33	211	104	93.8	403	13	288	57.7	204	367	23	196	63	108	287	<0.001
Starch	88	147	20.5	0	709	33	282	33.8	0	571	13	78.8	17.7	0	190	23	204	24.1	21	436	<0.001
CP	88	124	35	44.7	247	33	139	30.9	65.5	178	13	150	29.9	113	196	23	156	29.3	122	214	<0.001
Digestible OM	88	622	90.9	458	852	33	674	109	461	789	13	629	76.9	512	736	23	706	42.5	605	776	<0.001
Digestible NDF	88	292	102	94.9	474	33	215	82.7	97.3	400	13	268	34.7	212	323	23	235	84.1	121	403	<0.001
Digestible CP	88	74.3	29.8	4.9	135	33	89.3	32.8	5	130	13	101	26.9	65.6	139	23	107	30.2	61.1	166	<0.001
Rumen fermentable OM	88	503	40.4	413	586	33	481	31	421	586	13	488	38.5	450	544	23	541	44.1	436	631	<0.001
Rumen digestible NDF	88	263	92	85.4	427	33	193	74.3	87.7	360	13	241	31.1	190	291	23	211	75.8	109	363	<0.001
Rumen fermentable CP	88	72.9	23.9	27.6	128	33	75.2	22.9	28.5	119	13	105	31.1	58.7	149	23	102	25.5	66.1	167	<0.001
INRA (2007) PDI	88	78.7	19.4	27.7	112	33	91.3	22	26.9	115	13	84.5	9.1	73.3	96.8	23	97.9	15.2	80.5	126	<0.001
ME (MJ/kg DM)	88	9.51	1.51	6.75	13.1	33	10.4	1.79	6.76	12.2	13	9.55	1.19	7.75	11.4	23	11.2	0.955	9.17	12.5	<0.001
Proportion of concentrate (g/100 g DM)	88	29.5	35.8	0	100	33	47.3	38	0	98.8	13	35.3	22.7	0	75	23	48.5	29.4	10	100	0.025
Intake (g/kg BW per day)																					
DM	88	21.2	7.96	0	41.5	33	20.6	7.79	8.21	38.8	13	19.5	7.41	0	29.6	23	26.4	6.4	19.1	36.9	0.014
Digestible OM	88	13.2	5.09	0	23	33	13.5	4.1	4.39	20.2	13	12.5	5.14	0	19.1	23	18.7	4.39	13.1	28.6	<0.001
Rumen fermentable OM	88	10.7	4.17	0	20.8	33	9.93	3.74	3.45	18.5	13	9.63	3.83	0	13.8	23	14.2	3.05	11.2	17.4	<0.001
Rumen digestible NDF	88	5.68	3.17	0	14.3	33	4.27	2.98	1.25	11.2	13	4.65	1.68	0	6.58	23	5.36	1.68	2.68	10.6	0.095
Starch	88	3.13	4.18	0	15.2	33	5.27	3.78	0	12.4	13	1.76	1.6	0	5	23	5.75	3.77	0.4	16.1	0.001
Energy balance (kJ/kg BW per day)	88	20.8	57.6	−158	109	33	43.9	49.5	−173	91.2	13	−46.4	51.4	−159	15.3	23	−2.4	39	−70.9	58.8	<0.001

Min = minimum value; Max = maximum value; *n*_t_ = number of treatments; OM = organic matter; ME = metabolizable energy.

a See the list of publications used in the meta-analysis in Supplementary Material S2.

b Fasting included.

c Dairy cattle were between 11 and 240 days in milk.

d Dietary composition and intake calculated by additivity according to INRA Feed Tables (INRA, 2007); PDI = protein digestible in the intestine, defined as the minimum between protein digestible in the intestine when energy is limiting and protein digestible in the intestine when nitrogen is limiting.

The potential contribution of precursors to net hepatic release of glucose averaged 51.4 ± 19.9%, 40.4 ± 27.3% and 23.5 ± 23.8% for propionate, *α*-amino-N and l-lactate, respectively, with large differences between physiological statuses (Table 2). Average values showed a general excess of C from total precursors, except in gestating and lactating animals. The amount of mobilized glycerol was estimated at 1.59 ± 1.26 mmol/kg BW per h and the average amount of alanine mobilized from muscles was estimated at 0.092 ± 0.073 mmol C/kg BW per h (*n* = 32, Table 2). Accuracy of these calculated values could not be evaluated due to the lack of published data. Out of the analytical methods tested, only the pAH analysis significantly influenced the relationships (Supplementary Material S3 and Table S2).

**Table 2:** Description of the reported nutrient arterial concentrations, net hepatic fluxes^a^ and potential contribution to gluconeogenesis in ruminants used for the meta-analyses according to physiological status

	Non-productive adults^b^					Growing animals					Gestating animals					Lactation^c^					Effect^d^
	*n*_t_	Mean	SD	Min	Max	*n*_t_	Mean	SD	Min	Max	*n*_t_	Mean	SD	Min	Max	*n*_t_	Mean	SD	Min	Max	*P-*value
Arterial concentration (mM)																					
Propionate	25	0.0355	0.0347	0.012	0.16	14	0.0586	0.0147	0.031	0.08	5	0.046	0.013	0.032	0.061	6	0.075	0.027	0.039	0.096	0.011
*α*-amino-N	45	4.35	0.89	2.86	7.19	25	2.95	0.419	2.23	3.7	3	2.71	0.042	0	4.09	2	3.02	0.0071	3.02	3.03	<0.001
l-lactate	28	0.803	0.372	0.34	1.42	25	0.574	0.148	0.364	1.1	5	0.492	0.133	0.361	0.65	11	0.418	0.194	0.194	0.756	<0.001
Glucose	48	3.27	0.535	1.89	4.56	25	4.42	0.535	3.3	5.49	5	3.57	0.106	3.47	3.74	14	3.52	0.439	2.73	4.2	<0.001
Insulin, μIU/L	7	38.9	23	18.4	71.9	6	31.9	11.8	21.3	51.5	2	14.5	2.17	13	16	4	9.43	3.32	6.31	13.8	0.045
Net portal appearance (mmol/kg BW per h)																					
Propionate	37	0.656	0.417	0.085	2.11	16	0.8	0.21	0.47	1.16	11	0.64	0.18	0.382	0.447	11	1.16	0.29	0.827	1.89	<0.001
*α*-amino-N	61	0.597	0.408	−0.01	2.71	29	0.354	0.198	0.029	0.95	3	0.251	0.229	0	0.447	2	0.636	0.106	0.561	0.711	<0.001
l-lactate	41	0.274	0.166	0.084	0.767	25	0.203	0.083	0.117	0.399	5	0.117	0.0217	0.091	0.141	15	0.236	0.0816	0.074	0.363	0.031
Glucose	61	−0.091	0.102	−0.373	0.168	25	−0.05	0.098	−0.234	0.16	5	−0.026	0.0193	−0.044	0	23	−0.026	0.136	−0.166	0.352	<0.001
Net hepatic flux (mmol/kg BW per h)																					
Propionate	35	−0.562	0.292	−1.1	−0.085	16	−0.705	0.183	−1.034	−0.409	11	−0.591	0.186	−0.892	−0.254	11	−1.08	0.279	−1.69	−0.757	<0.001
*α*-amino-N	63	−0.422	0.203	−0.923	0.436	23	−0.378	0.376	−1.3	−0.033	3	−0.23	0.237	−0.474	0	2	−0.274	0.086	−0.335	−0.213	<0.001
l-lactate	41	−0.356	0.346	−1.94	0.029	27	−0.189	0.131	−0.399	−0.008	5	−0.079	0.126	−0.297	0	17	−0.271	0.158	−0.48	−0.107	0.48
Glucose	69	0.67	0.31	0.245	1.74	27	0.551	0.161	0.317	0.975	5	0.34	0.193	0	0.463	23	0.995	0.313	0.467	1.405	<0.001
Potential contribution to gluconeogenesis (%)																					
Propionate	35	41.6	20.2	12.3	99.7	16	66.5	13.3	46.3	94.7	4	49.7	13.5	34	65.5	11	60.9	10.1	49.4	81.1	<0.001
*α*-amino-N	45	46.3	29.3	0	180.2	17	30.2	16.7	6.94	82.1	2	19.2	27.2	0	38.4	2	16.4	1.36	15.4	17.3	0.061
l-lactate	41	31.9	30.8	0	149	27	17.6	11.8	0.608	44.3	4	12.6	18.4	0	39.8	17	15.2	10	0	42.8	0.018
Estimated^e^ mobilized nutrients (mmol/kg BW per day)																					
Mobilized glycerol	18	1.68	1.26	0.058	4.31	0	ND	ND	ND	ND	2	2.91	2.55	1.11	4.72	8	1.066	0.64	0.092	1.93	0.159
Mobilized alanine	18	0.097	0.073	0.0034	0.249	0	ND	ND	ND	ND	2	0.169	0.148	0.064	0.273	8	0.062	0.04	0.0053	0.112	0.159

Min = minimum value; Max = maximum value; *n*_t_ = number of treatments; ND = not determinated.

a A positive value indicates a net release; a negative value indicates a net uptake.

b Fasting included.

c The dairy cattle were between 11 and 240 days in milk.

d Physiological status effect.

e Values are reported only for animals in negative energy balance.

### Net hepatic uptake of propionate, l-lactate and *α*-amino-N

#### Comparison of models using net portal appearance or afferent fluxes as predictor

**Figure 1:**
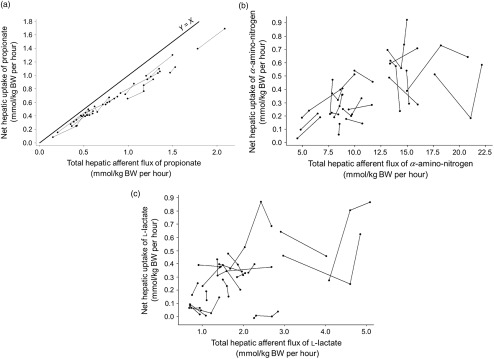
Within-study relationships between the net hepatic uptake of propionate (a), *α*-amino-N (b) and l-lactate (c) and their total hepatic afferent flux in ruminants. The propionate (a), *α*-amino-N (b) and l-lactate (c) datasets were used.

**Figure 2:**
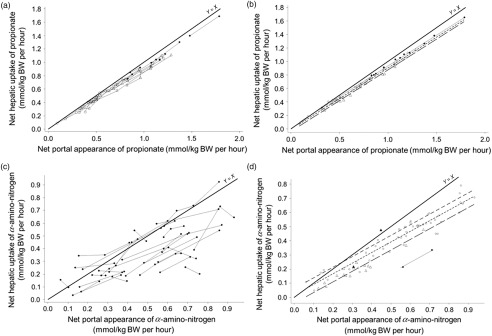
Within-study relationships between net hepatic uptake and net portal appearance of propionate (a for raw data and b for adjusted model) and *α*-amino-N (c for raw data and d for adjusted model) in ruminants. The propionate (a, b) and *α*-amino-N (c, d) datasets were used. Adjusted models are shown for non-productive adults (◯), growing animals (△), lactating cattle (•) and gestating animals (▴).

**Figure 3:**
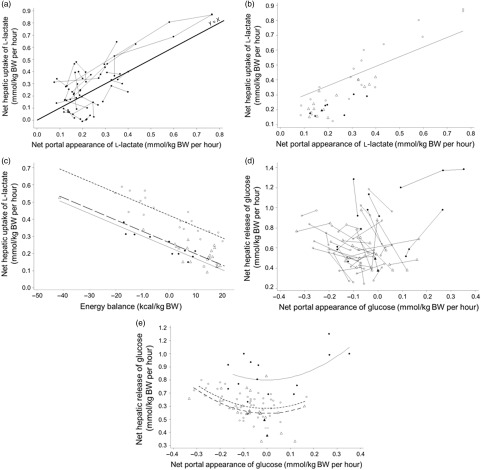
Within-study relationships between net hepatic uptake of l-lactate and net portal appearance of l-lactate (a for raw data, and b for adjusted model), and energy balance (c for adjusted model); and between net hepatic release of glucose and its net portal appearance (d for raw data, e for adjusted model) in ruminants. The l-lactate (a, b, c) and glucose (d, e) datasets were used. Adjusted models are shown for non-productive adults (◯), growing animals (△), lactating cattle (•) and gestating animals (▴).

Quantitatively, NPA contributed to 85.8 ± 6.1% (propionate), 4.8 ± 1.7% (*α*-amino-N) and 15.4 ± 6.3% (l-lactate) of the hepatic afferent flux. For propionate, net hepatic uptake was similarly related to NPA and afferent flux. By contrast, for total amino acids and l-lactate net hepatic uptake was better related to NPA than to afferent flux (Figure 1 *v.* Figures 2 and 3; Supplementary Material Table S4). As a result, the subsequent meta-analysis considered NPA as the X variable.

#### Models

The net hepatic uptake of propionate, *α*-amino-N and l-lactate increased linearly with their net absorption in the portal vein (*P* < 0.01, Table 3). For propionate and *α*-amino-N, average intercepts were not significantly different from zero. The linear slopes suggested that for 1 mmol/kg BW per h appearing in portal vein, 0.910, 0.749 and 0.474 mmol/kg BW per h of propionate, *α*-amino-N (corresponding to a slope of 0.536 for total amino acids) and l-lactate were taken up by the liver, respectively.

**Table 3:** Response equations of the net hepatic fluxes (NHFs, mmol/kg BW per h) of propionate (C3), *α*-amino-nitrogen (*α*N) and l-lactate to variations in their net portal appearance (NPA, mmol/kg BW per h) and energy balance (EB, kcal/kg BW per day) in ruminants

Model number	*Y*	Number^a^				Equation	Adjustment		Effect^b^		Interfering factors^c^
		*n*_exp_	*n*_*α*i_	*n*_t_	*n*_r_		RMSE	Adjusted *R*^2^	PHY	*X* * PHY	LSMeans
Net hepatic fluxes											
1	Propionate	27	2	69	0	−0.0035 ± 0.022^NS^ − 0.910 ± 0.028*** × NPA-C3	0.028	0.992	<0.001	NS	EB**; starch intake*
2	α-N	26	11	65	3	0.0413 ± 0.0353^NS^ − 0.749 ± 0.068*** × NPA-*α*N	0.062	0.898	0.001	NS	+CP^†^; +PDI*;-NHF-C3*; +NHF-gluc*
3	l-Lactate	25	14	66	7	−0.0151 ± 0.0430** − 0.474 ± 0.20* × NPA-l-lactate	0.067	0.892	NS	0.058	+NPA-gluc**; -NHF-C3*; +PDI*; +dOM**; +ME*
4		18	4	43	0	−0.2195 ± 0.0621** + 0.218 ± 0.080** × NPA-C3 − 1.029 ± 0.096 × NPA-l-lactate	0.060	0.916	NS	NS	None
5		25	17	54	4	−0.3090 ± 0.0091*** + 0.00648 ± 0.00142*** × EB	0.057	0.908	0.001	NS	+NPA-gluc**; -NPA-*α*N**

NS = not significant (*P* > 0.10); RMSE = residual means square error; *X* = explanatory variable; LSMeans = least-squares means; EB = energy balance; PDI = protein digestible in the intestine; gluc = glucose; dOM = dietary concentrations of digestible organic matter; ME = metabolizable energy.

All models were based on reported measured fluxes.

^†^*P* < 0.10; *P < 0.05; **P < 0.01; ***P < 0.001.

a *n*_exp_: number of experimental groups in the model; *n*_*α*i_: number of experimental groups with *α*i (intercept for the experimental group i) significantly different from zero; *n*_t_: number of treatments in the model; *n*_r_: number of treatments rejected from the model (outliers).

b PHY: physiological status, effects of physiological status were detected on the intercept (Model 1, Δ = −0.0005 (NS), 0.032, −0.026 (*P* = 0.06), −0.0061 (NS); Model 2, Δ = −0.0584, −0.0009 (NS), 0,161, −0,104; Model 5, Δ = −0.1149, 0.0442, 0.0707, ND), on the slope (Model 3, Δ = −0.507, 0.333 (NS), −0.174 (NS), ND) for the non-productive adults, growing animals, lactation and gestation, respectively.

c No interfering factors were observed on individual slopes and residuals; these are interfering factors on the LSMeans: the minus sign indicates a negative effect on the net hepatic flux; the plus sign indicates a positive effect on the net hepatic flux.

#### Interfering factors

Table 3 reports the significant interfering factors and Supplementary Material Table S5 reports the linear relationships between LSMeans and interfering factors. For propionate (model 1, Figure 2a and b) and at similar NPA of propionate (LSMeans effect), the net hepatic uptake increased with the energy balance of the animals (*P* < 0.01) and starch intake (*P* ≤ 0.05). For *α*-amino-N (model 2, Figure 2c and d) and at similar NPA of *α*-amino-N, net hepatic uptake increased (*P* > 0.05) with dietary N concentration (CP and protein digestible in the intestine) and net hepatic release of glucose (*P* ≤ 0.05), and decreased with the net hepatic uptake of propionate (*P* ≤ 0.05). When introduced in the model as additional covariates, none of those factors significantly improved the fit. For l-lactate (model 3, Figure 3a and b) and at similar NPA of l-lactate, the net hepatic uptake was significantly affected by propionate uptake. Inclusion of propionate uptake as a covariate significantly improved the model, removing all interfering factors (model 4). To account for the role of lactate to recycle C, energy balance was also tested as an X variable, but did not improve the fit and showed significant interfering factors (model 5, Figure 3c).

### Net hepatic release of glucose

#### Influence of the net portal absorption of glucose

The net hepatic release of glucose was quadratically related (*P* < 0.05) to its NPA (model 6, Table 4; Figure 3d and e), indicating that the net release of glucose decreased when NPA of glucose increased from −0.334 up to 0 mmol/kg BW per h, and increased thereafter.

**Table 4:** Response equations of the net hepatic release of glucose (*Y* variable) to the following *X* variables: net portal appearance (NPA) and net hepatic flux (NHF) of propionate (C3), total amino acids (tAA), l-lactate, glucose and sum of precursors (mmol C/kg BW per h) in ruminants

Model number	Predictors		Numbers^a^				Intercept		Slope for *X*1		Slope for *X*2				Effects^b^		Interfering factor^c^
	*X*1	*X*2	*n*_exp_	*n*_*α*i_	*n*_t_	*n*_r_	*α*	SD	*β*1	SD	*β*2	SD	RMSE	*R*^2^_adj_	PHY	*X* * PHY	LSMeans
6	NPA-glucose	(NPA-glucose)^2^	42	16	93	0	0.591***	0.028	NS		2.0096*	0.826	0.13	0.725	<0.001	NS	None
7	NPA-C3		26	8	57	0	2.259***	0.486	0.714***	0.185	NS	ND	0.532	0.848	<0.001	NS	+NHF-BHBA**; +NHF- *α*N**
8	NHF-C3		27	6	59	0	1.856***	0.66	−0.923*	0.426	NS	ND	0.494	0.869	NS	0.058	None
9	NPA-tAA^d^		26	12	61	2	0.309^NS^	0.733	1.288***	0.254	NS	ND	0.405	0.899	<0.001	<0.001	None
10	NHF-tAA^d^		21	8	52	0	2.134**	0.691	−1.163**	0.404	NS	ND	0.694	0.75	0.008	0.006	None
11	NPA-l-lactate		34	10	72	0	2.403***	0.424	2.062**	0.66	NS	ND	0.794	0.698	<0.001	NS	+ME intake *
12	NHF-l-lactate		35	15	76	1	4.214***	0.207	0.537^†^	0.284	NS	ND	0.654	0.835	<0.001	NS	None
13	NPA-precursors^e^		39	14	89	6	−0.708^NS^	0.727	0.809***	0.112	NS	ND	0.424	0.896	<0.001	<0.001	NHF-BHBA*
14	NPA-precursors^e^	NPA-glucose	34	12	82	0	1.364***	0.334	0.636**	0.094	−0.613**	0.168	0.503	0.855	0.001	NS	None
15	NHF-precursors^e^		30	16	71	6	−0.384^NS^	0.531	−0.905***	0.096	NS	ND	0.375	0.925	<0.001	<0.001	None

ND = not determined; NS = not significant (*P* > 0.10); RMSE = residual means square error; LSMeans = least-squares means; BHBA = beta-hydroxybutyrate; ME = metabolizable energy; *α*N = *α*-amino-nitrogen.

^†^*P* < 0.10; *P < 0.05; **P < 0.01; ***P < 0.001.

a *n*_exp_: number of experimental groups in the model; *n*_*α*i_: number of experimental groups with *α*i significantly different from zero; *n*_t_: number of treatments in the model; *n*_r_: number of treatments rejected from the model (outliers).

b PHY = physiological state effect, effects of physiological status were detected on the intercept (Model 6, Δ = −0.007 (NS), −0.045 (NS), 0.210, −0.158; Model 7, Δ = 0.276, −0.773, 0.497; Model 9, Δ = 2.336, 0.317 (NS), −3.023 (NS), 0.369 (NS); Model 10, Δ = 1.533, −0.583 (NS), −0.457, −0.492 (NS); Model 11, Δ = −0.907, −0.092 (NS), 1.512, −0.512 (NS); Model 12, Δ = −0.286, −0.345, 2.047, −1.416; Model 13, Δ = 2.676, 0.984 (NS), −3.661, ND; Model 14, Δ = −0.199 (*P* = 0.1), −0.341, 0.540, ND; Model 15, Δ = 2.478, −0.862, −1.615, ND), on the slope (Model 8, Δ = 0.237 (NS), 1.308, −0.934 (NS), −0.610 (NS); Model 9, Δ = −0.908, −0.059 (NS), 1.342, −0.374 (NS); Model 10, Δ = 1.157, 0.247 (NS), −2.070, 0.666 (NS); Model 13, Δ = −0.511, −0.129 (NS), 0.640, ND; Model 15, Δ = 0.573, −0.289, −0.285, ND) for the non-productive adults, growing animals, lactation and gestation, respectively.

c No interfering factors were observed on individual slopes and residuals: the minus sign indicates a negative effect on the net hepatic flux; the plus sign indicates a positive effect on the net hepatic flux.

d tAA fluxes calculating from *α*-amino-nitrogen fluxes according to Martineau *et al.* (2009).

e Percentage of estimated precursors : 30.6%, 29.6% and 31%, respectively, for models 13, 14 and 15.

#### Influence of the hepatic uptake of each precursor

Generally, the net hepatic release of glucose increased significantly and linearly with the availability of each precursor considered separately, in the portal vein (Table 4, models 7, 9, 11) and taken up by the liver (Table 4, models 8, 10, 12). Intercepts were generally significantly different from zero, indicating that glucose release also depends on other factors. At zero NPA of total amino acids, the uptake of total amino acids by the liver was higher with productive animals (growing and lactating) *v.* non-productive animals (*P* < 0.001, Table 4, model 9). At similar NPA of propionate (model 7, LSMeans effect, *P* = 0.001) the net hepatic release of glucose increased when the hepatic uptake of amino acids decreased, and when the hepatic release of β-hydroxybutyrate increased. No improvement of fit was gained when these factors were added in the model.

#### Influence of the hepatic uptake of the sum of precursors

**Figure 4:**
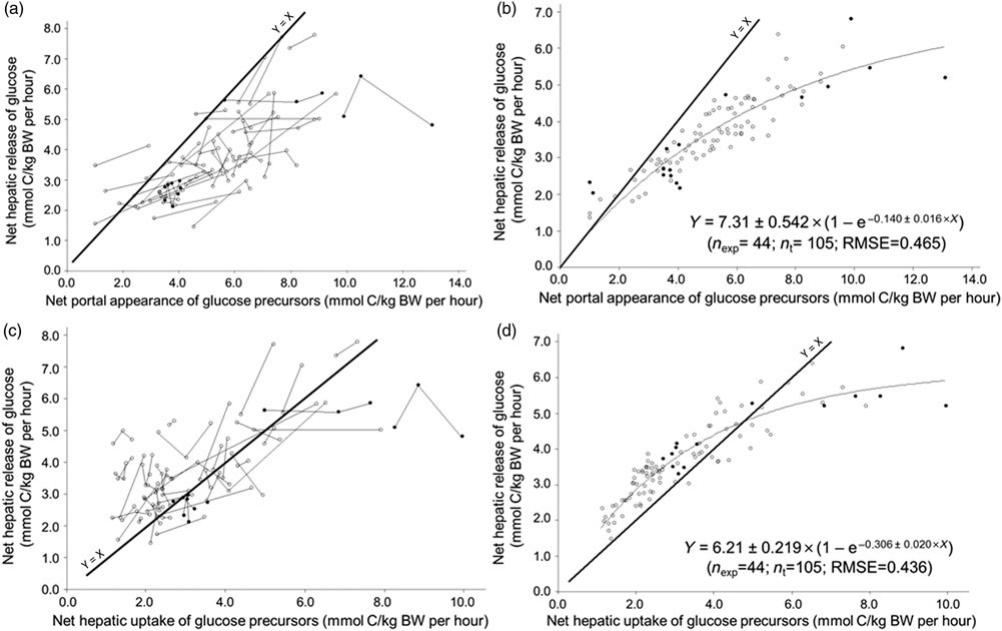
Within-study curvilinear relationships between net hepatic release of glucose and availability of summed precursors expressed as net portal appearance (a for raw data, b for adjusted model) and as net hepatic uptake (c for raw data, d for adjusted model) in ruminants. The combined all-precursor and propionic infusion datasets were used. Number of experimental groups (*n*_exp_) and number of treatments (*n*_t_) are given.

When the all-precursor and the propionic infusion datasets were combined to evaluate the glucose response over a wide range of precursor supply, glucose release was curvilinearly related to precursor availability (Figure 4). In this combined dataset, the NPA of summed precursors ranged from 1.003 to 13.05 mmol C/kg BW per h (Figure 4a and b), while net hepatic uptake ranged from 1.098 to 9.944 mmol C/kg BW per h (Figure 4c and d). At similar precursor availability (LSMeans effect on both models), glucose release was increased when the net hepatic release of *β*-hydroxybutyrate increased (*P* < 0.05).

**Figure 5:**
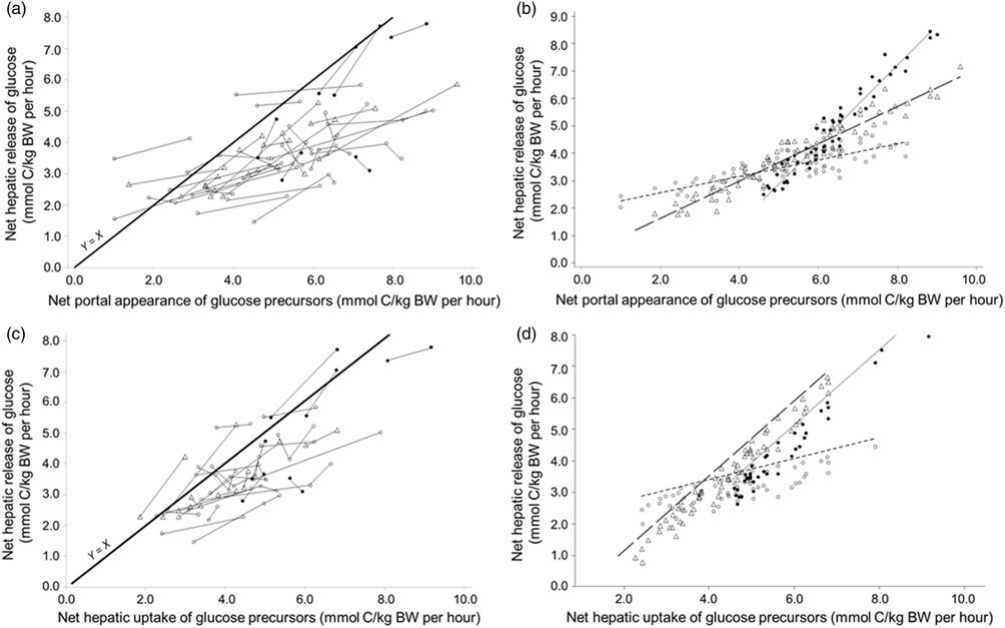
Within-study linear relationships between net hepatic release of glucose and availability of summed precursors expressed as net portal appearance (a for raw data, b for adjusted model) and as net hepatic uptake (c for raw data, d for adjusted model) in ruminants. The all-precursor dataset was used. Adjusted models are shown for non-productive adults (◯), growing animals (△), lactating cattle (•) and gestating animals (▴).

Another meta-analysis was conducted on the all-precursor dataset only, excluding the propionate infusion studies, to explore the more linear portion of the relationship. The NPA of summed precursors ranged from 0.994 to 9.69 mmol C/kg BW per h and was closer to the physiological range. A linear model (quadratic model not significant, results not shown) was applied and the net hepatic release of glucose increased by 0.809 mmol C/kg BW per h for each increment of the NPA of summed precursors (Table 4, model 13; Figure 5a and b) with a good adjustment (RMSE represented 13% of average release of glucose by the liver). The intercept was not different from zero (*P* < 0.001) suggesting no net glucose release in the absence of precursor supply. Both the intercept and the slope were significantly affected by physiological status indicating a higher net release of glucose from the same net portal absorption of precursors for lactating animals. At similar NPA of precursors (LSMeans effect), glucose release was increased when hepatic emission of *β*-hydroxybutyrate increased. Interestingly when the NPA of glucose was added as covariate in the model (Table 4, model 14), it had a negative impact (slope effect) of the net hepatic release of glucose. Finally, the net hepatic release of glucose increased linearly by 0.905 mmol C/kg BW per h for each increment in net hepatic uptake of precursors (in mmol C/kg BW per h; Table 4, model 15, Figure 5c and d), with a good adjustment (RMSE represented 10% of average release of glucose by the liver). Both the intercept and the slope were significantly affected by physiological status, indicating a higher conversion rate of precursors into glucose for productive animals (growing and lactating) *v.* non-productive adults.

### Net hepatic insulin fluxes

The insulin dataset was limited to *n* = 19 treatments. Arterial concentrations ranged from 6.3 to 71.9 μUI/ml and increased (intra-study slope) by 3.04 ± 0.36 μUI/ml per g DMI per kg BW per day (*P* = 0.004). The NPA of insulin also increased with intake (expressed in g/kg BW per day) by 1.31 ± 0.25 μUI/kg BW per h (*P* = 0.001, *n*_t_ = 17) per g DM intake, by 9.81 ± 1.61 μUI/kg BW per h per g of starch digested in the small intestine (*P* = 0.01, *n*_t_ = 8) and by 8.02 ± 1.43 μUI/kg BW per h per g NDF digested in the rumen (*P* = 0.001, *n*_t_ = 17). Moreover, the NPA of insulin tended to increase with the portal – arterial concentration difference (in mM) of propionate (slope = 44.48 ± 20.16; *P* = 0.11, *n*_t_ = 8) and acetate (slope = 10.82 ± 4.43; *P* = 0.09, *n*_t_ = 8), but not glucose (*P* = 0.23). The net hepatic uptake of insulin increased with the NPA of insulin (slope = 0.49 ± 0.14, *P* = 0.01, *n*_t_ = 17), the portal – arterial concentration difference of propionate (slope = 57.94 ± 6.60, *P* = 0.01, *n*_t_ = 8) and with the NPA of glucose (slope = 27.65 ± 9.96, *P* = 0.05, *n*_t_ = 16).

## Discussion

The present work substantiates the use of the NPA, instead of total afferent fluxes, as the predictor for the net hepatic uptake of propionate, *α*-amino-N and l-lactate in a database like the one used in the current study where within-study variations were mainly caused by dietary changes and where lactating cow data were obtained at mid-lactation. The higher the contribution of NPA to the afferent flux, the lower the difference in fit between the two predictors. Obviously, the relevance of using the NPA as predictor depends on the nutrient and on the range of nutritional situations under study. As a result, for propionate, *α*-amino-N and to a lower extent for l-lactate their net hepatic uptakes follow mass action laws, with NPA as the main statistical driving force. The statistical driving force may differ from the biological one. This is especially the case for the amino acid fluxes, which are more accurate than the *α*-amino-N ones, and for animals that are not representative of this dataset. Indeed, when comparing cows pre- and post-calving, it was clear that increased net portal absorption of amino acids post-calving accompanying increased DMI was not related to increased hepatic net removal, the latter declining in response to decreased arterial amino acids concentrations related to the initiation of lactation (Doepel *et al.*, 2009). Also as a consequence of this statistical choice, endogenous l-lactate availability from mobilized tissues, as in early lactation, is not accounted for.

The within-study approach which was applied here focuses on incremental responses. It is testing the hypotheses that marginal changes in the *X* variables are inducing marginal changes in the *Y* variables of interest. This same strategy had also been applied to predict the net hepatic release of ketogenic nutrients by Loncke *et al.* (2015). As a result, the study of the incremental supply of nutrients, which is primarily driven by daily intake and the related gut metabolism, is dissociated from the baseline levels of nutrient fluxes, which also depend on animal characteristics. It is compatible with the static models of feed evaluation systems which calculate daily rations for animals, irrespective of the amount of nutrients already circulating in the animal from previous rations as developed in Ortigues-Marty *et al.* (2019).

### Domain of application of the models

Empirical prediction models apply to a strict range of validity. Because available data were balanced between cattle and sheep, models apply to both species. Species or physiological status significantly affected models of the present work, but as in Loncke *et al.* (2015) it was not always possible to evaluate whether it was a strict species or physiological status effect, or if there was some confounding with nutritional conditions. These effects are discussed below for each nutrient. In all cases, using physiological status as a covariate was more discriminant than using species as in Loncke *et al.* (2015) for ketogenic nutrients. This covariate also encompasses differences in the level of production. As for other models developed from the same Flora database (Loncke *et al.*, 2009b and 2015), models apply to animals with intakes up to 41 g DMI/kg BW per day of diets varying from 0 to 100 g concentrate/100 g DM. They do not account for any feed additive effects (buffers, essential oils, ionophores, etc.) nor for lipid supplementation.

Out of the analytical methods considered, only pAH analysis and hence blood flow evaluation had a significant quantitative influence on the relationships (Supplementary Material S3). Acetylation of pAH was shown to result in overestimation of nutrient release and underestimation of nutrient uptake by Larsen and Kristensen (2013) and Rodriguez-Lopez *et al.* (2014), except for amino acids uptake which was reduced. When considering marginal rates instead of absolute values, both release and uptake rates were overestimated by liver acetylation of pAH. Despite these effects, we decided not to correct nutrient fluxes in order not to introduce further uncertainties to the data besides those inherent to the measurements (Rodriguez-Lopez *et al.*, 2016).

### Hepatic uptake of individual precursors

#### Propionate

Results confirm the high hepatic uptake of propionate (Armentano, 1992). In the propionate dataset, no data exceeded the extraction capacity of the liver, propionate supply remained below levels obtained with infusions (e.g. Berthelot *et al.*, 2002) and portal propionate concentrations remained inferior to 1 mM. The intercept, not different from zero, was consistent with zero uptake in the absence of ruminal fermentations. The lowest hepatic uptake of propionate noted in growing animals was compatible with the known inhibitory effect of butyrate on propionate extraction (Berthelot *et al.*, 2002), as in the dataset hepatic supply and uptake of butyrate were elevated (Loncke *et al.*, 2015).

#### l-Lactate

Hepatic uptake of l-lactate was related to two different types of predictors. First, amounts of l-lactate and propionate appearing in the portal vein, which reflect dietary nutrient supply and synthesis of l-lactate from glucose in gut tissues (van der Walt *et al.*, 1983) that increases at high propionate levels (Harmon *et al.* 1991). Considering NPA of propionate as an additional predictor significantly improved the model (model 4 *v.* 3) and removed all interfering factors. We interpreted this improvement as the result of high propionate availability in the current dataset, and subsequently an increased hepatic conversion of propionate into l-lactate as shown *in vitro* by Demigné *et al.* (1991).

Second, the energy balance which reflects the nutritional status of the animal and the recycling of C through the Cori cycle when animals are in tissue mobilization (Reynolds, 2005). This predictor is relevant, even if there was no gain in precision probably because of uncertainties of estimations. It did not fully eliminate the effect of physiological status suggesting an effect of physiological status per se, as reported, for example, in transition cows with an increase in l-lactate removal by the liver and in its potential contribution to liver glucose synthesis immediately after calving (Benson *et al.*, 2002; Reynolds *et al.*, 2003).

#### *α*-amino-N

The statistical mass action law which describes the impact of the portal appearance of *α*-amino-N on its net hepatic uptake (also observed by Lescoat *et al.* (1996) and Reynolds (2006)) strictly applies to *α*-amino-N as a proxy of the summed amino acids and does not apply to individual amino acids whose uptake is more related to their total afferent flux (Doepel *et al.*, 2009). Interestingly, the physiological and probably the underlying nutritional status of the animal (species, diet composition and intake, and level of production) influenced the baseline uptake (intercept) and not the marginal uptake rate (slope). Baseline net hepatic uptake (intercept) was higher in sheep than in cattle, probably reflecting lower production levels and lower tissue amino acid requirements (Blouin *et al.*, 2002; Reynolds, 2006). In a meta-analysis with a different dataset from the same Flora database, Martineau *et al.* (2011) attributed the higher recovery of ingested nitrogen in the portal vein of sheep *v.* cattle to a strict species effect. It was assumed to result from lower amino acid losses through portal-drained viscera metabolism in sheep or from a more efficient nitrogen recycling at lower nitrogen intake (Martineau *et al.*, 2011 and 2014). Our results also showed that the average level of *α*-amino-N hepatic uptake (LSMeans) can be modified by the dietary N concentration, availability in propionate and hepatic glucose release. Direct experimental evidence was reported by Kraft *et al.* (2011) who showed that a N deficient diet significantly limited the net hepatic uptake of *α*-amino-N, as a sparing mechanism, without modifying net glucose release (Loncke *et al.*, 2009a).

### Hepatic conversion of precursors into glucose

An interesting outcome of this work is to show that the net hepatic release of glucose increases linearly with the availability of summed precursors (portal appearance or hepatic uptake) up to approximately 6 mmol C/kg BW per h, beyond which the conversion rate decreases. In the present dataset, the highest levels of precursor supply were met by intra-gastric infusion of individual nutrient (e.g. Majdoub *et al.*, 2003). Whether it is the high level of precursor supply or the imbalanced nutrient supply (as in Kraft *et al.*, 2011 with N deficient diets) which is responsible for this plateau would remain to be confirmed. This reduced efficiency of hepatic conversion of precursors into glucose suggests a supply of precursors in excess of glucose demand.

When considering a smaller range of summed precursor (i.e. excluding propionate infusion studies only) availability, the precursor C was converted to glucose at a rate (slope) ranging from 0. 64 to 0.81 fold its net portal supply or 0.91 fold its hepatic uptake. Conversion rates remained below 1 and were subject to the influence of physiological status probably indicating some regulation in the conversion of precursors into glucose by the demand for glucose as discussed by Lapierre *et al.* (2010). At equal NPA of precursors (LSMean effect), the net release of glucose by the liver was higher for dairy cattle and lower for non-productive and growing animals, confirming the higher whole body glucose turnover rates observed for high *v.* low producing animals fed at similar metabolizable energy intake (Wilson *et al.*, 1983; Ortigues-Marty *et al.*, 2003). The impact of physiological status was also noted on the conversion rate of propionate into glucose (slope) which was highest for dairy cattle and lowest for growing animals (effect on intercept) as previously pointed out (Reynolds *et al.*, 2003).

Our results showed that for a same supply of precursors, the conversion of precursor C into glucose (LSMeans effect on model 13) and the conversion of portally absorbed propionate into glucose (model 7) increase when β-hydroxybutyrate uptake increases. They suggest increased glucose release when body reserves are mobilized as suggested by Loncke *et al.* (2009b), that is, animals in negative energy balance are more efficient in synthesizing glucose from precursors than when in positive energy balance. An increased conversion rate of precursors into glucose can be hypothesized because no increased contribution of endogenous precursors is expected as discussed above and reviewed by Larsen and Kristensen (2013).

Current results clearly show that bypass starch will limit the hepatic release of glucose reducing the conversion of precursors into glucose. The general relationship with precursor supply as well as the curvilinear relationship with NPA of glucose clearly demonstrated it. The supply of 117 g of bypass starch, equivalent to an NPA of 100 mmol glucose, will decrease the net hepatic release of glucose by 61 mmol of glucose, implying a substitution rate of 61% (model 14). Two mechanisms may be considered. First, starch intake also provides additional propionate, and the higher the propionate supply, the lower the propionate conversion rate into glucose as discussed above and the lower the net hepatic uptake of other precursors. Second, insulin/glucagon regulations might be involved. At negative NPA of glucose, that is, when bypass starch intake is null or low (lower than ≈1.7 g/kg BW per day, or lower than ≈200 g starch/kg DM, Loncke *et al.*, 2009c), precursor (mostly propionate; Majdoub *et al.*, 2003) absorption enhances hepatic insulin clearance and thereby the inhibitory effect of insulin on gluconeogenesis, possibly by reducing the entry of precursors in the gluconeogenesis pathway (Aschenbach *et al.*, 2010). At positive NPA of glucose, direct effects of insulin at hepatic level are unlikely because hepatic extraction of insulin was not modified by the NPA of glucose. Instead regulations may involve changes in the insulin/glucagon ratio (Brown and Allen, 2013). This ratio is curvilinearly affected by starch intake, and it decreases as a result of increased glucagonemia when the dietary starch concentration is higher than 160 g/kg DM (Garnsworthy *et al.*, 2008). In lactating dairy cows, glucose supply to the animal will indeed increase with glucose absorption (Reynolds, 2005).

### Insulin

Insulin is an important regulator of hepatic metabolism of glucose and its precursors. Unfortunately, insulin net flux data were scarce, and it was a deliberate choice not to fully develop quantitative response models. It was also not possible to include it as additional covariate. However, the within-study response equations were sufficiently reproducible to draw useful conclusions. Individual nutrients have different insulino-secretory effects. Insulin secretion, reflected by its NPA, increased with the intake level and absorption (portal – arterial concentration difference) of propionate and acetate. The insulino-secretory effect of propionate shown experimentally by intra-abomasal (Casse *et al.*, 1994) or intra-ruminal (Majdoub *et al.*, 2003) infusions was almost seven times higher than that of acetate. The latter may not be a physiological regulator (Harmon, 1992). Furthermore, the sites of action of insulin may vary in relative terms with the nature of absorbed nutrients. On average, 49 ± 14% of the NPA of insulin was taken up by the liver. But the hepatic clearance of insulin increased with the availability in volatile fatty acids (NPA), more specifically in propionate and butyrate (portal – arterial concentration difference), confirming experimental evidence for propionate (Majdoub *et al.*, 2003). This phenomenon is specific to the ruminants (Harmon *et al.*, 1991; Harmon, 1992). It suggests a proportionally greater action of insulin on liver functions, as compared to other tissues, when propionate and butyrate availability is high. By contrast, a positive NPA of glucose had no influence on the hepatic clearance of insulin, explaining why peripheral insulinaemia is more responsive to glucose than to volatile fatty acids (propionate) absorption and suggesting a proportionally greater action of insulin on tissues such as the adipose tissues and the muscles, as compared to the liver. This confirms that physiological levels of volatile fatty acids, especially propionate, and glucose differentially regulate insulin secretion, hepatic clearance and peripheral insulinaemia (Harmon, 1992; Gualdron-Duarte and Allen, 2018).

In conclusion, glucose synthesis in the ruminant liver is tightly regulated. It does not strictly respond to precursor availability according to a single mass action law. Individual precursors are taken up by the liver in proportion of their availability but their uptake varies with the availability of other precursors. Also the rate of conversion of precursors into glucose is not fixed. Net glucose release varies curvilinearly with supply and uptake of precursors by the liver, suggesting a law of diminishing returns when precursor availability is in excess of glucose requirements. The linear portion of the response provides quantitative indications of the impacts of glucose demand, net portal absorption of glucose and negative energy balance on the net hepatic glucose release. These relationships can be linked with the predictions of portal absorption of nutrients from intake and dietary characteristics, and thereby provide indications of glucose synthesis from dietary and animal characteristics.

## Software and data repository resources

The Flora database has been deposited at the Agence pour la Protection des Programmes, 249 rue de Crimée, 75019 Paris, France, IDDN.FR.001.380017.000.R.P.2010.000.10300. Authors are open to collaborations.

## Supplementary material

For supplementary material accompanying this paper visit https://doi.org/10.1017/S1751731119003410. click here to view supplementary material
