# Supplementary material for: Net hepatic release of glucose from precursor supply in ruminants: a meta-analysis
Source: Animal. 2020 Jan 23;14(7):1422–37. doi: 10.1017/S1751731119003410 (PMC7301244; doi:10.1017/S1751731119003410)
Supplement: Supplementary file 1 [file S1751731119003410sup001.docx]

Net hepatic release of glucose from precursor supply in ruminants: a meta-analysis

 C. Loncke, P. Nozière, J. Vernet, H. Lapierre**,** L. Bahloul, M. Al-Jammas, D. Sauvant, I. Ortigues-Marty.

*Animal*: an International Journal in Animal Biosciences

**SUPPLEMENTARY MATERIALS**

**Supplementary Material S1. Terminology on net nutrient fluxes**

Afferent flux: Flux of nutrient supplied to the liver via the portal vein and the hepatic artery

= (nutrient concentration in portal venous blood/plasma x portal venous blood/plasma flow) + (arterial nutrient concentration x hepatic arterial blood/plasma flow)

Efferent flux: Flux of nutrient released by the liver via the hepatic vein

= nutrient concentration in hepatic venous blood/plasma x hepatic venous blood/plasma flow

Net hepatic flux = efferent hepatic flux – afferent hepatic flux

The terminology ‘net hepatic uptake’ refers to a positive net hepatic flux, ie. afferent flux > efferent flux.

The terminology ‘net hepatic release’ refers to a negative net hepatic flux, ie. afferent flux < efferent flux.

**Supplementary Material S2. List of references used in the meta-analysis**

Atkinson RL, Toone CD, Robinson TJ, Harmon DL and Ludden PA 2007. Effects of supplemental ruminally degradable protein versus increasing amounts of supplemental ruminally undegradable protein on nitrogen retention, apparent digestibility, and nutrient flux across visceral tissues in lambs fed low-quality forage. Journal of Animal Science 85, 3331-3339.

Baird GD, Lomax MA, Symonds HW and Shaw SR 1980. Net hepatic and splanchnic metabolism of lactate, pyruvate and propionate in dairy cows in vivo in relation to lactation and nutrient supply. Biochemical Journal 186, 47-57.

Berthelot V, Pierzynowski SG, Sauvant D and Kristensen NB 2002. Hepatic metabolism of propionate and methylmalonate in growing lambs. Livestock Production Science 74, 33-43.

Bergman, EN, Katz ML, Kaufman CF 1970. Quantitative aspects of hepatic and portal glucose metabolism and turn-over in sheep. American Journal of Physiology 219, 785-793.

Bohnert DW, Larson BT, Lewis S, Richards CJ, Swanson KC, Harmon DL and Mitchell GE 1999. Net nutrient flux in visceral tissues of lambs fed diets differing in supplemental nitrogen source. Journal of Animal Science 77, 2545-2553.

Branco AF, Mouro GF, Harmon DL, Rigolon LP, Zeoula LM, Maia FJ and Coneglian SM 2004. Fontes de proteina, ingestao de alimentos e floxo esplancnico de nutrients emo vinos. Revista Brasileira de Zootecnia 33, 444-452.

Brockman RP 1987. Effect of exercise on net hepatic uptake of lactate, pyruvate, alanine, and glycerol in sheep. Canadian Journal of Physiology and Pharmacology 65, 2065-2070.

Burrin DG, Ferrell CL, Eisemann JH and Britton RA 1991. Level of nutrition and splanchnic metabolite flux in young lambs. Journal of Animal Science 69, 1082-1091.

De Visser HA, Valk H, Klop A, Van der Meulen J, Bakker JGM and Huntington GB 1997. Nutrient fluxes in splanchnic tissue of dairy cows: Influence of grass quality. Journal of Dairy Science 80, 1666-1673.

De Visser HA, Klop A, Van der Meulen J and Van Vuuren AM 1998. Influence of maturity of grass silage and flaked corn starch on the production and metabolism of volatile fatty acids in dairy cows. Journal of Dairy Science 81, 1028-1035.

Eisemann JH and Nienaber JA 1990. Tissue and whole-body oxygen uptake in fed and fasted steers. British Journal of Nutrition 64, 399-411.

Eisemann JH, Huntington GB and Catherman DR 1996. Patterns of nutrient interchange and oxygen use among portal-drained viscera, liver and hindquarters of beef steers from 235 to 525 kg body weight. Journal of Animal Science 74, 1812-1831.

Ferrell CL, Kreikemeier KK and Freetly HC 1999. The effect of supplemental energy, nitrogen, and protein on feed intake, digestibility, and nitrogen flux across the gut and liver in sheep fed low-quality forage. Journal of Animal Science 77, 3353-3364.

Ferrell CL, Freetly HC, Goetsch AL and Kreikemeier KK 2001. The effect of dietary nitrogen and protein on feed intake, nutrient digestibility, and nitrogen flux across the portal-drained viscera and liver of sheep consuming high-concentrate diets ad libitum. Journal of Animal Science 79, 1322-1328.

Freetly HC and Klindt J 1996. Changes in gut and liver glucose, lactate, insulin, and oxygen flux in mature ewes during mesenteric or abdominal vena cava glucose infusion. Journal of Nutrition 126, 924-932.

Freetly HC and Ferrell CL 1998. Net flux of glucose, lactate, volatile fatty acids, and nitrogen metabolites across the portal drained viscera and liver of pregnant ewes. Journal of Animal Science 76, 3133–3145.

Fukuma TK, Tangiguchi K and Obitsu T 2005. Evaluation of fishmeal supplement with net nitrogen flux by the portal-drained viscera and the liver in mature sheep. Asian-Australian Journal of Animal Science 18, 1251-1291.

Goetsch AL and Ferrell CL. 1995. Effect of dietary maize level on net flux across splanchnic tissues of oxygen and nutrients in wethers consuming ad-libitum different forages. Animal Science 61, 43-55.

Goetsch AL, Ferrell CL and Freetly HC 1994. Effect of different supplements on splanchnic oxygen consumption and net fluxes of nutrients in sheep consuming bromegrass (Bromus inermis) hay ad libitum. British Journal of Nutrition 72, 701-712.

Goetsch AL, Patil AR, Galloway DL, Kouakou B, Wang ZS, Park KK and Rossi JE 1997a. Net flux of nutrients across splanchnic tissues in wethers consuming grasses of different sources and physical forms ad libitum. British Journal of Nutrition 77, 769-78.

Goetsch AL, Patil AR, Wang ZS, Park KK, Galloway DL, Rossi JE and Kouakou B 1997b. Net flux of nutrients across splanchnic tissues in wethers consuming bermudagrass or ryegrass-wheat hay supplemented with rumen undegradable protein. Small Ruminant Research 25, 119-128.

Goetsch AL, Patil AR, Galloway DL, Wang ZS, Kouakou B, Park KK and Rossi JE 1997c. Oxygen consumption by splanchnic tissues in wethers consuming ad libitum differents proportions of bermudagrass and ryegrass-wheat. Archives of Animal Nutrition 50, 1-11.

Goetsch AL, Patil AR, Wang ZS, Park KK, Galloway DL, Rossi JE and Kouakou B 1997d. Net flux of nutrients across splanchnic tissues in wethers consuming grass hay with or without corn and alfalfa. Animal Feed Science and Technology 66, 271-282.

Harmon DL, Gross KL, Kreikemeier KK, Coffey KP, Avery TB and Klindt J. 1991. Effects of feeding endophyte-infected fescue hay on portal and hepatic nutrient flux in steers. Journal of Animal Science 69, 1223-1231.

Heitmann RN, Sensenig SC, Reynolds CK, Fernandez JM and Dawes DJ 1986. Changes in energy metabolite and regulatory hormone concentrations and net fluxes across splanchnic and peripheral tissues in fed and progressively fasted ewes. Journal of Nutrition 116, 2516-2524.

Huntington GB, Zetina E, Whitt JM and Potts W 1996. Effects of dietary concentrate level on nutrient absorption, liver metabolism, and urea kinetics of beef steers fed isonitrogenous and isoenergetic diets. Journal of Animal Science 74, 908-916.

Katz ML and Bergman EN 1969. Hepatic and portal metabolism of glucose, free fatty acids, and ketone bodies in the sheep. American Journal of Physiology 216, 953-960.

Krehbiel CR, Ferrell CL and Freetly HC 1998. Effects of frequency of supplementation on dry matter intake and net portal and hepatic flux of nutrients in mature ewes that consume low-quality forage. Journal of Animal Science 76, 2464-2473.

Kristensen NB and Harmon DL 2004a. Splanchnic metabolism of volatile fatty acids absorbed from the washed reticulorumen of steers. Journal of Animal Science 82, 2033-2042.

Kristensen NB and Harmon DL 2004b. Effect of increasing ruminal butyrate absorption on splanchnic metabolism of volatile fatty acids absorbed from the washed reticulorumen of steers. Journal of Animal Science 82, 3549-3559.

Kristensen NB and Harmon DL 2005. Effects of adding valerate, caproate, and heptanoate to ruminal buffers on splanchnic metabolism in steers under washed-rumen conditions. Journal of Animal Science 83, 1899-1907.

Kristensen NB, Storm A, Raun BML, Rojen BA and Harmon DL 2007. Metabolism of silage alcohols in lactating dairy cows. Journal of Dairy Science 90, 1364-1377.

Lapierre H, Reynolds CK, Elsasser TH, Gaudreau P, Brazeau P and Tyrrell HF 1992. Effects of growth hormone-releasing factor and feed-intake on energy-metabolism in growing beef steers - net hormone metabolism by portal-drained viscera and liver. Journal of Animal Science 70, 742-751.

Linington MJ, Meyer JHF and van der Walt JG 1998. Ruminal VFA production rates, whole body metabolite kinetics and blood hormone concentrations in sheep fed high- and low-fibre diets. South African Journal of Animal Science 28, 82-98.

Lozano O, Theurer CB, Alio A, Huber JT, Delgado-Elorduy A, Cuneo P, DeYoung D, Sadik M and Swingle RS 2000. Net absorption and hepatic metabolism of glucose, L-lactate, and volatile fatty acids by steers fed diets containing sorghum grain processed as dry-rolled or steam-flaked at different densities. Journal of Animal Science 78, 1364-1371.

Majdoub L., Vermorel M and Ortigues-Marty I 2003a. Ryegrass-based diet and barley supplementation: Partition of energy-yielding nutrients among splanchnic tissues and hind limbs in finishing lambs. Journal of Animal Science 81, 1068-1079.

Majdoub L, Vermorel M and Ortigues-Marty I. 2003b. Intraruminal propionate supplementation modifies hindlimb energy metabolism without changing the splanchnic release of glucose in growing lambs. The British Journal of Nutrition 89, 39-50.

Maltby SA, Lomax MA, Beever DE and Pippard CJ 1992. The effect of increased ammonia and amino acid supply on post prandial portal-drained viscera and hepatic metabolism in growing steers fed maize silage. In 12th Symposium on Energy Metabolism of Farm Animals (eds C Wenk and C Boessinger), pp 20-22. EAAP Publication, Zurich, Switzerland.

Ortigues I, Visseiche AL and Durand D 1994. Adaptation to undernutrition in ewes: Net nutrient fluxes across the portal drained viscera, the liver and the hindquarters. In 13th Symposium on Energy Metabolism of Farm Animals (eds JF Aguilera), pp 89-92. EAAP Publication, Granada, Spain.

Park KK, Goetsch AL, Johnson ZB and Rossi JE 1997. Temporal net flux pattern of nutrients across splanchnic tissues in wethers consuming different forages. Small Ruminant Research 25, 107-118.

Patil AR, Goetsch AL, Park KK, Kouakou B and Galloway DL, Johnson ZB 1995a. Influence of grass source on net flux of nutrients across splanchnic tissues in sheep with restricted intake. Archives of Animal Nutrition 48, 257-269.

Patil AR, Goetsch AL, Park KK, Kouakou B, Galloway DL, West CP and Johnson ZB 1995b. Net flux of nutrients across splanchnic tissues in sheep fed tropical vs temperate grass hay of moderate or low qualities. Livestock Production Science 43, 49-61.

Patil AR, Goetsch AL, Park KK, Kouakou B, Galloway DL and Johnson Z. 1996. Influence of grass source and legume level on net flux of nutrients across splanchnic tissues in sheep. Small Ruminant Research 22, 111-122.

Reynolds CK and Tyrrell HF 1991. Effects of mesenteric vein L-alanine infusion on liver metabolism in beef heifers fed on diets differing in forage - concentrate ratio. British Journal of Nutrition 66, 437-450.

Reynolds CK, Tyrrell HF and Reynolds PJ 1991. Effects of diet forage-to-concentrate ratio and intake on energy metabolism in growing beef heifers: Net nutrient metabolism by visceral tissues. Journal of Nutrition 121,1004-1015.

Reynolds CK, Huntington GB, Tyrrell HF and Reynolds PJ 1988a. Net portal-drained visceral and hepatic metabolism of glucose, L-lactate and nitrogenous compounds in lactating Holstein cows. Journal of Dairy Science 71, 1803-1812.

Reynolds CK, Huntington GB, Tyrrell HF and Reynolds PJ 1988b. Net metabolism of volatile fatty acids, D-beta-hydroxybutyrate, nonesterified fatty acids, and blood gasses by portal-drained viscera and liver of lactating Holstein cows. Journal of Dairy Science 71, 2395-2405.

Reynolds CK, Aikman PC, Lupoli B, Humphries DJ and Beever DE 2003. Splanchnic metabolism of dairy cows during the transition from late gestation through early lactation. Journal of Dairy Science 86, 1201-1217.

Røjen BA, Raun BML, Lund P and Kristensen NB 2004. Effect of supplement strategy on splanchnic net fluxes of ammonia and urea in dairy cows fed fresh grass. Journal of Animal Feed Science 13, 347-350.

Theurer CB, Huntington GB, Huber JT, Swingle RS and Moore JA 2002. Net absorption and utilization of nitrogenous compounds across ruminal, intestinal, and hepatic tissues of growing beef steers fed dry-rolled or steam-flaked sorghum grain. Journal of Animal Science 80, 525-532.

Thompson GE, Bassett JM and Bell AW 1978a. The effects of feeding and acute cold exposure on the visceral release of volatile fatty acids, estimated hepatic uptake of propionate and release of glucose, and plasma insulin concentration in sheep. British Journal of Nutrition 39, 219-226.

Thompson GE, Manson W, Clarke PL and Bell AW 1978b. Acute cold exposure and the metabolism of glucose and some of its precursors in the liver of the fed and fasted sheep. Quarterly Journal of Experimental Physiology 63, 189-199.

Wieghart M, Slepetis R, Elliot JM and Smith DF 1986. Glucose absorption and hepatic gluconeogenesis in dairy cows fed diets varying in forage content. Journal of Nutrition 116, 839-850.

**Supplementary Table S1.** *Calculation of endogenous metabolites mobilised when ruminants are in negative energy balance*

| Calculation of energy balance^§1^ |
| --- |
| ME requirements for maintenance (**ME_m_**, kJ/kg BW ^0.75^ per day)  = 481.5 for dairy cattle and beef cattle  = 439.6 for dry and gestating cattle and for sheep  When ME intake < ME_m_ :  Energy Balance (**EB,** kJ /kg BW ^0.75^ per day) = (ME intake – ME_m_) × k_m_  When ME intake > ME_m_ :  Expected NE for production (**NEP**, kJ /kg BW ^0.75^ per day) = (ME intake - ME_m_) × k  Energy Balance (EB)  = expected NEP, for growing animals  = expected NEP – observed NEP, for lactating or gestating animals  Mobilized energy (kJ/kg BW ^0.75^ per day) = EB, when EB < 0 |
| Calculation of mobilized alanine^§2^ |
| Mobilized proteins (g/kg BW ^0.75^ per day) = (mobilized energy x 0.293) / 5.6  Mobilized alanine (moles of carbon/kg BW ^0.75^ per day) = (mobilized proteins / 89.1) x 4 |
| Calculation of mobilized glycerol^§3^ |
| Mobilized fat (g/kg BW ^0.75^ per day) = (mobilized energy x 3.89) / 9.2  Mobilized glycerol (moles of carbon/kg BW ^0.75^ per day) = [ (mobilized fat x 0.105) / 92] x 3 |

Table modified from Loncke C, Nozière P, Bahloul L, Vernet J, Lapierre H, Sauvant D and Ortigues-Marty I. 2015. Empirical prediction of net splanchnic release of ketogenic nutrients, acetate, butyrate and β-hydroxybutyrate in ruminants: A meta-analysis. Animal 9, 449-463. (reprinted with permission)

^§1^ ME= metabolisable energy; NE = net energy; GE = gross energy; k_m_ = 0.287 × ME/GE + 2.32 ; k = k_l_ = 0.249 × ME/GE + 1.94 for lactation and moderate growth or k = k_g_ = 0.13 for gestation ; observed NEP for lactation = 3.094 MJ/L milk per day, assuming a milk fat content at 4% (except in the 5 publications which reported milk fat content ranging from 3.6% to 4.5%), and NEP for gestation = 17.15 MJ/day for Holstein cows, 6.70 MJ/day for Dorset sheep and 10.7 MJ/day for Lacaune sheep. All values are based on INRA (2007)

^§2^ with 0.07 being the proportion of proteins in mobilized energy, 5.6 the energy density of proteins (MJ/g), 89.1 the molar mass of alanine (g/mol) and 4 the number of carbons in one mole of alanine (mole/mole)

^§3^ with 0.93 being the proportion of fat in mobilized energy, 9.2 the energy density of fat (MJ/g), 0.105 the proportion of glycerol in mobilized fat assuming that body fat is composed of 100% triglycerides and that one mole of triglyceride comprises 1 mole of glycerol and 3 moles of fatty acids (of 887 g/mole of average molar mass), 92 the molar mass of glycerol (g/mol), and 3 the number of carbons in one mole of glycerol (mole/mole)

**Supplementary Material S3.** **Influence of analytical methods on models**

Attention was paid to the methods used to determine net hepatic fluxes to ensure that all results could be combined in the meta-analysis. Methodological aspects considered were the selection of the matrix, blood or plasma, and of the analytical method for the analysis of metabolites, as well as the presence *vs*. absence of a deacetylation step in the analysis of para-aminohippuric acid used to determine blood or plasma flows.

*Measurement of metabolites on blood vs plasma.* In the eligible dataset, concentrations were mainly determined on blood (n = 49 and 4 for propionate, n = 76 and 25 for α-amino-N, n = 60 and 30 for L-lactate and n = 58 and 60 for glucose, in blood and plasma respectively). No results on total or individual amino acid fluxes were available. For propionate (data not shown) and α-amino-N (Martineau *et al*., 2009), no difference had been observed whether fluxes were measured in plasma or blood. For L-lactate, differences between blood and plasma fluxes depend on the nutritional status of the animals (Aufrère, 1979). For glucose, net portal appearance of glucose tended to be overestimated by plasma measurements when it was low, but underestimated up to 25 % with high intakes in ewes (Nozière *et al*., 1998). But no systematic correction factor exists for L-lactate and glucose. Hence all plasma and blood results were pooled. The lack of gross bias between plasma and blood results was only checked by graphical examination.

*Analytical methods to determine metabolite concentrations.* Analytical methods used to measure glucose (oxi-peroxydase), α-amino-N (ninhydrine) and L-lactate (L-lactate dehydrogenase) were similar among publications. Similarly, all but one (solvent extraction) selected publications indicated that propionate was analysed after an extraction by ion exchange resins. Consequently, no correction was applied to account for differences in analytical methods. It was checked that all blood or plasma concentrations of nutrients were superior to the analytical limits of quantification (Ortigues *et al*., 2003) determined in our laboratory (limit of quantification = 1.13 mM for glucose, 0.19 mM for propionate and 0.32 mM for α-amino-N, not defined for L-lactate). All data met this analytical reliability criterion.

*Impact of para-aminohippuric acid determination method*. Hepatic blood flows were measured using the para-aminohippuric acid (**pAH**) down-stream dilution method. 86 % of all publications did not correct for the incomplete recovery of pAH across the liver, demonstrated in sheep by Katz and Bergman (1969) and in cows by Kristensen *et al*. (2009) and Rodriguez-Lopez *et al.* (2014). To evaluate the potential impact of this methodological error, it was tested whether correction of net hepatic fluxes for incomplete marker recovery modified the prediction equations. Net fluxes were recalculated assuming that the hepatic arterial blood/plasma flow represented 10% of hepatic venous blood/plasma flow as measured when pAH is deacetylated before analysis (Rodriguez-Lopez *et al.*, 2014). This correction could only be applied when publications reported blood flow and nutrient arterial concentration data in addition to net fluxes. It was the case in 77, 68, 67 and 88 % of the publications used in the models of glucose, propionate, lactate and α-amino-N, respectively.

Correcting net flux values for incomplete recovery of pAH could only be done for a limited number of publications. This limited dataset did not span over the whole meta-design and reduced the proportion of data on lactation and gestation, explaining why some uncorrected response equations established on this partial dataset (Supplementary Table S2) were significantly different from those reported in Tables 4 and 5. Correcting net flux values for pAH acetylation did not modify net hepatic uptake of propionate, increased net uptakes of L-lactate and reduced that of α-amino-N and glucose. Values averaged 0.679±0.053 *vs.* 0.680±0.053, 0.276±0.031 *vs.* 0.294±0.030, 0.393±0.033 *vs.* 0.353±0.025, and 0.712±0.037 *vs.* 0.695±0.038 mmol/ kg BW per hour, for uncorrected and corrected fluxes, in the same respective order of nutrient. The marginal rates of nutrient uptake by the liver were significantly reduced for L-lactate and α-amino-N only, as well as the marginal rate of glucose release from available precursors. Intercept values remained not significantly different from zero, changes in their numerical values reflected changes in net fluxes reported above.

Aufrère J 1979. Relations inter-organes et capitation hépatique des principaux substrats de la néoglucogenèse et de la cétogenèse chez le rat. Influence des facteurs nutritionnels et du jeûne. PhD thesis, Université de Clermont II, Clermont Ferrand, France.

**Supplementary Table S2** *Response models of net hepatic fluxes (NHF, mmol/kg BW per hour) of propionate (C3), L-lactate, α-amino-N (*αN*) and glucose (mmol C/kg BW per hour) after correction of hepatic blood flows for incomplete para-aminohippuric (pAH) recovery in ruminants. Since corrections could not be applied to the full dataset, uncorrected and corrected values are shown for the sub-datasets used*

|  |  | pAH acid uncorrected values | | | pAH corrected values | | |
| --- | --- | --- | --- | --- | --- | --- | --- |
| Models | n_exp_ | Equations | RMSE | *R^2^_adj_* |  | RMSE | *R^2^_adj_* |
| NHF-C3 | 21 | 0.0646^NSa^±0.0345 - 0.9896**^b^±0.0443 x NPA-C3 | 0.0275 | 0.99 | 0.0541^NSc^±0.0291 - 0.9812***^c^±0.0374 x NPA-C3 | 0.0232 | 0.99 |
| NHF-L-lactate | 12 | -0.2235^NSa^±0.1067 + 0.2322**^a^±0.1313 x NPA-C3  - 1.0677^***a^±0.1244 x NPA-L-lactate | 0.0613 | 0.91 | -0.1873^NSc^±0.0817 + 0.1379**^d^±0.1006 x NPA-C3  - 0.9904^***d^±0.0954 x NPA-L-lactate | 0.0469 | 0.94 |
| NHF-αN | 23 | -0.0431^NSb^±0.0482 - 0.5819**^b^±0.0879 x NPA-αN | 0.0805 | 0.90 | -0.0891^NSd^±0.0483 - 0.4615*^d^±0.088 x NPA- αN | 0.0808 | 0.83 |
| NHF-glucose | 30 | -0.7407^NSa^±0.8820 + 0.7971^**a^±0.1233 x NPA-prec | 0.4352 | 0.90 | 0.495^NSc^±1.496 + 0.6824**^c^±0.2092 x NPA-prec | 0.738 | 0.81 |

n_exp_ = number of experimental groups in the model; RMSE = Residual means square error; *R^2^_adj_* = adjusted *R^2^*; NS: non significantly different from zero; * *P*<0.05; ** *P*<0.01; *** *P*<0.001; NPA = net portal appearance; prec = glucose precursors

^a^ No significant difference with the same parameter calculated from the whole dataset

^b^ Significant difference with the same parameter calculated from the whole dataset

^c^ No significant difference with the same parameter calculated from the pAH-uncorrected sub-dataset (*P*>0.05)

^d^ Significant difference with the same parameter calculated from the pAH-uncorrected sub-dataset (*P*<0.05)

**Supplementary Material S4.** **Influence of animal profile**

*Influence of physiological status*

Non-productive adults (56% of the data) were characterised by DM intake ranging from fasting to 41 g/kg BW. Growing animals (21% of the data) and lactating cows were fed diets rich in concentrate (47-48%, P<0.03 compared to other physiological statuses). All lactating animals (15% of the data) were dairy cows between 11 and 240 days in milk (13 treatments for the first 80 days in milk, and 10 for 120 to 240 days in milk). They were fed the highest levels of DM intake (*P*=0.049) compared to animals in other physiological status. Data on gestating animals (8% of the data; all in late gestation, ≤ 2 months before calving) were limited, but sufficient to be included in some analyses.

*Influence of animal species*

For each nutrient, the distribution of treatments was similar between cattle and sheep and between females and males (data not shown). Among available data, cattle and sheep had similar average DM intake per kg BW (*P*=0.47) but diets fed to sheep had different composition with lower proportions of concentrate (*P*<0.001) and lower digestibility (*P*<0.001) compared with cattle. Consequently, dietary intakes differed between cattle and sheep for all constituents except for dietary rumen fermentable organic matter intake (*P*=0.60) (data not shown).

**Supplementary Table S3.a** *Description of diets used for the meta-analyses^1^ of net hepatic fluxes of propionate, L-lactate, α-amino-nitrogen and glucose according to species (cattle and sheep)*

|  | Cattle | | | | |  | Sheep | | | | | |  | Species effect |
| --- | --- | --- | --- | --- | --- | --- | --- | --- | --- | --- | --- | --- | --- | --- |
|  | n_t_ | mean | SD | Min | Max |  | n_t_ | mean | SD | Min | | Max |  | P value |
| Dietary composition^2^ (g/kg DM) | | | | | | | | | | | | | | |
| Crude fiber | 62 | 171 | 71.7 | 74.6 | 371 |  | 95 | 273 | 83.7 | 39.5 | 404 | |  | < 0.001 |
| NDF | 62 | 352 | 122 | 170 | 710 |  | 95 | 522 | 137 | 133 | 712 | |  | < 0.001 |
| ADF | 62 | 188 | 75.9 | 87.7 | 403 |  | 95 | 296 | 86.7 | 43.8 | 393 | |  | < 0.001 |
| Starch | 62 | 52.6 | 36.3 | 0.0 | 110 |  | 95 | 21.3 | 30.0 | 0.0 | 106 | |  | < 0.001 |
| CP | 62 | 152 | 29.9 | 85.0 | 247 |  | 95 | 122 | 33.2 | 44.7 | 181 | |  | < 0.001 |
| Digestible OM | 62 | 710 | 65.7 | 478 | 793 |  | 95 | 604 | 85.3 | 458 | 852 | |  | < 0.001 |
| Digestible NDF | 62 | 216 | 83.2 | 97.3 | 403 |  | 95 | 297 | 92.1 | 94.9 | 474 | |  | < 0.001 |
| Digestible CP | 62 | 102 | 27.0 | 39.0 | 166 |  | 95 | 72.9 | 30.8 | 4.90 | 135 | |  | < 0.001 |
| Rumen fermentable OM | 62 | 508 | 41.4 | 436 | 631 |  | 95 | 500 | 43.7 | 413 | 586 | |  | 0.238 |
| Rumen digestible NDF | 62 | 195 | 74.8 | 87.7 | 363 |  | 95 | 268 | 82.9 | 85.4 | 427 | |  | < 0.001 |
| Rumen fermentable CP | 62 | 92.1 | 26.8 | 45.9 | 167 |  | 95 | 72.5 | 25.1 | 27.6 | 128 | |  | < 0.001 |
| PDI | 62 | 95.6 | 14.9 | 54.3 | 126 |  | 95 | 77.5 | 19.8 | 26.9 | 111.5 | |  | < 0.001 |
| ME (MJ/kg DM) | 62 | 11.1 | 1.15 | 7.03 | 12.5 |  | 95 | 9.20 | 1.42 | 6.75 | 13.1 | |  | < 0.001 |
| Proportion of concentrate  (g/100 g DM) | 62 | 54.1 | 30.4 | 0.0 | 100 |  | 95 | 25.0 | 33.6 | 0.0 | 100 | |  | < 0.001 |
|  |  |  |  |  |  |  |  |  |  |  |  | |  |  |
| Intake (g/kg BW per day) |  |  |  |  |  |  |  |  |  |  |  | |  |  |
| DM | 62 | 21.0 | 5.90 | 0.0 | 36.9 |  | 95 | 22.1 | 8.92 | 0.0 | 47.5 | |  | 0.378 |
| Digestible OM | 62 | 14.9 | 4.51 | 0.0 | 28.6 |  | 95 | 13.4 | 5.47 | 0.0 | 30.0 | |  | < 0.001 |
| Rumen fermentable OM | 62 | 10.7 | 3.26 | 0.0 | 17.4 |  | 95 | 11.1 | 4.61 | 0.0 | 25.4 | |  | 0.540 |
| Rumen digestible NDF | 62 | 4.07 | 1.74 | 0.0 | 7.53 |  | 95 | 6.03 | 3.23 | 0.0 | 14.3 | |  | < 0.001 |
| Starch | 62 | 5.60 | 3.68 | 0.0 | 16.1 |  | 95 | 2.70 | 3.90 | 0.0 | 15.2 | |  | < 0.001 |
|  |  |  |  |  |  |  |  |  |  |  |  | |  |  |
| Energy balance  (kJ/kg BW per day) | 62 | 29.9 | 43.0 | -70.9 | 88.9 |  | 95 | 11.2 | 61.0 | -173 | 109.4 | |  | < 0.001 |

Min = minimum value; Max = maximum value; n_t_ = number of treatments

^1^ see Supplementary Material S1

^2^ Dietary composition and intake calculated by additivity according to INRA Feed Tables (INRA, 2007); DM = dry matter; OM = organic matter; ME = metabolizable energy; PDI = protein digestible in the intestine; BW = body weight.

**Supplementary Table S3.b** *Description of arterial concentrations, net portal appearance, net hepatic fluxes^a^ and estimated potential contribution to neoglucognenesis of propionate, L-lactate, α-amino-N (α-N) and glucose used for the meta analysis^b^ of net hepatic fluxes of propionate, L-lactate, α-amino-N and glucose according to species (cattle and sheep)*

|  | Cattle | | | | |  | Sheep | | | | |  | Species Effect |
| --- | --- | --- | --- | --- | --- | --- | --- | --- | --- | --- | --- | --- | --- |
|  | n_t_ | mean | SD | Min | Max |  | n_t_ | mean | SD | Min | Max |  | *P-*value |
| Arterial concentration (mM) | | | | | | | | | | | | | |
| Propionate | 26 | 0.0608 | 0.0186 | 0.031 | 0.096 |  | 24 | 0.0334 | 0.0348 | 0.012 | 0.160 |  | 0.001 |
| α-amino-N | 24 | 3.29 | 1.07 | 2.27 | 7.19 |  | 51 | 4.017 | 1.017 | 0.0 | 6.10 |  | 0.006 |
| L-lactate | 40 | 0.493 | 0.145 | 0.200 | 0.756 |  | 29 | 0.842 | 0.344 | 0.340 | 1.42 |  | < 0.001 |
| Glucose | 43 | 4.035 | 0.645 | 2.73 | 5.49 |  | 49 | 3.29 | 0.546 | 1.89 | 4.56 |  | < 0.001 |
| Insulin (μUI/L) | 12 | 21.5 | 13.7 | 6.31 | 51.47 |  | 7 | 38.9 | 23.0 | 18.4 | 71.9 |  | 0.520 |
|  |  |  |  |  |  |  |  |  |  |  |  |  |  |
| Net portal appearance (mmol/kg BW per hour) | | | | | | | | | | | | | |
| Propionate | 40 | 0.904 | 0.285 | 0.447 | 1.78 |  | 35 | 0.589 | 0.40 | 0.085 | 2.11 |  | < 0.001 |
| α-amino-N | 28 | 0.358 | 0.174 | 0.091 | 0.711 |  | 67 | 0.577 | 0.405 | 0.0 | 2.71 |  | 0.007 |
| L-lactate | 44 | 0.250 | 0.091 | 0.074 | 0.403 |  | 42 | 0.250 | 0.167 | 0.084 | 0.767 |  | 0.41 |
| Glucose | 52 | -0.022 | 0.118 | -0.234 | 0.352 |  | 62 | -0.084 | 0.107 | -0.373 | 0.168 |  | 0.004 |
|  |  |  |  |  |  |  |  |  |  |  |  |  |  |
| Net hepatic flux (mmol/kg BW per hour) | | | | | | | | | | | | | |
| Propionate | 34 | -0.827 | 0.269 | -1.69 | -0.409 |  | 33 | -0.494 | 0.256 | -1.10 | -0.085 |  | < 0.001 |
| α-amino-N | 22 | -0.215 | 0.105 | -0.513 | -0.033 |  | 69 | -0.461 | 0.263 | -1.30 | 0.436 |  | < 0.001 |
| L-lactate | 46 | -0.236 | 0.292 | -1.94 | -0.107 |  | 44 | -0.314 | 0.237 | -0.870 | 0.029 |  | 0.168 |
| Glucose | 52 | 0.763 | 0.327 | 0.317 | 1.40 |  | 72 | 0.639 | 0.312 | 0.0 | 1.74 |  | 0.034 |
|  |  |  |  |  |  |  |  |  |  |  |  |  |  |
| Estimated potential contribution to neoglucogenesis (%) | | | | | | | | | | | | | |
| Propionate | 34 | 61.5 | 11.8 | 44.5 | 94.7 |  | 35 | 44.8 | 22.4 | 12.3 | 99.7 |  | < 0.001 |
| α-amino-N | 28 | 27.4 | 9.55 | 14.2 | 43.6 |  | 39 | 48.1 | 26.7 | 0.0 | 179 |  | 0.004 |
| L-lactate | 16 | 18.2 | 13.3 | 1.23 | 85.4 |  | 49 | 34.8 | 29.5 | 0.10 | 149 |  | 0.001 |

Min = minimum value; Max = maximum value; n_t_ = number of treatments

^a^ a positive value indicates a net release; a negative value indicates a net uptake

^b^ See Supplementary Material S1.

**Supplementary Material S5.** **Comparison of net hepatic uptake of nutrients predicted using net portal appearance of total afferent fluxes as the predictor**

**Supplementary Table S4.** *Response equations of the net hepatic fluxes (mmol/kg BW per hour) of propionate, α-amino-nitrogen, and L-lactate to variations in their net portal appearance (NPA, mmol/kg BW per hour) or total hepatic afferent flux (THAF, mmol/kg BW per hour) in ruminants*

| Y | Number | |  | Equation |  | Adjustment | |
| --- | --- | --- | --- | --- | --- | --- | --- |
|  | n_exp_ | n_t_ |  |  |  | RMSE | Adjusted R² |
| Propionate | 27 | 69 |  | 0.0024 ± 0.021^NS^ - 0.913 ± 0.027***× NPA-propionate |  | 0.027 | 0.992 |
|  | 19 | 48 |  | 0.091 ± 0.042* - 0.887 ± 0.049***× THAF-propionate |  | 0.003 | 0.990 |
| α-N | 30 | 85 |  | 0.0055 ± 0.033^NS^ - 0.749 ± 0.067 *** × NPA-α-amino-nitrogen |  | 0.062 | 0.898 |
|  | 20 | 50 |  | -0.025 ± 0.175^NS^ - 0.029 ± 0.157^†^ × THAF-α-amino-nitrogen |  | 0.127 | 0.609 |
| L-lactate | 25 | 66 |  | -0.066 ± 0.026* - 0.887 ± 0.105*** × NPA-L-lactate |  | 0.071 | 0.880 |
|  | 20 | 52 |  | -0.169 ± 0.097^†^ - 0.067 ± 0.051^NS^ × THAF-L-lactate |  | 0.126 | 0.677 |

NS: not significant (*P*>0.10); † *P*<0.10; * *P*<0.05; *** *P*<0.001; n_exp_: number of experimental groups in the model; n_t_: number of treatments in the model; RMSE = residual means square error

**Supplementary Material S6. LSMeans study**

**Supplementary Table S5.** Linear relationships between least square means (LSMeans, Y variable, mmol/h per kg BW) and interfering factors (X variable) detected in the models listed in Tables 3 and 4, in ruminants

| **Model number** | **Y variable, LSMeans of** | **X Variable** | **Intercept** | | **Linear term** | | **RMSE** | Adjusted **R²** |
| --- | --- | --- | --- | --- | --- | --- | --- | --- |
|  |  |  | **α** | **SD** | **β** | **SD** |  |  |
| 1 | NHF-C3 | EB  (kJ/d/kg BW) | -2,824*** | 0.251 | 0.0015** | 0.0005 | 0.113 | 0.245 |
| 1 | NHF-C3 | Starch intake (g/j/kg BW) | -0.684*** | 0.008 | 0.0004* | 0.0002 | 0.029 | 0.015 |
| 2 | NHF-aN | CP (g/kg DM) | -0.621*** | 0.119 | 0.0020* | 0.0009 | 0.114 | 0.132 |
| 2 | NHF-aN | PDIE (g/kg DM) | -0.725*** | 0.148 | 0.0039* | 0.0016 | 0.112 | 0.169 |
| 2 | NHF-aN | PDIN (g/kg DM) | -0.583*** | 0.001 | 0.0024* | 0.0011 | 0.115 | 0.122 |
| 2 | NHF-aN | NHF-glucose (mmol/h/kg BW) | -0.475*** | 0.061 | 0.2027* | 0.0918 | 0.109 | 0.156 |
| 3 | NHF-L-lactate | NHF-C3 (mmol/h/kg BW) | -0.470*** | 0.058 | -0.2349** | 0.0773 | 0.087 | 0.326 |
| 3 | NHF-L-lactate | NPA-glucose (mmol/h/kg BW) | -0.229*** | 0.030 | 0.6704* | 0.2646 | 0.112 | 0.184 |
| 3 | NHF-L-lactate | Starch intake (g/d/kg BW) | -0.353*** | 0.035 | 0.0168* | 0.0068 | 0.111 | 0.171 |
| 3 | NHF-L-lactate | ME (MJ /kg DM) | -3.230*** | 0.699 | 0.0002** | 0.0001 | 0.448 | 0.234 |
| 3 | NHF-L-lactate | DOM (g/kg DM) | -0.899*** | 0.181 | 0.0009** | 0.0003 | 0.102 | 0.299 |
| 5 | NHF-L-lactate | NPA-glucose (mmol/h/kg BW) | -0.191*** | 0.048 | 1.3195** | 0.4137 | 0.167 | 0.285 |
| 5 | NHF-L-lactate | NPA-N (mmol/h/kg BW) | -0.044 ^NS^ | 0.094** | -0.6733 | 0.2110 | 0.170 | 0.338 |
| 7 | NHF-glucose | NPA_N (mmol/h/kg BW) | 2.378*** | 0.585 | 3.6750** | 1.1770 | 0.866 | 0.368 |
| 7 | NHF-glucose | NHF-BHB (mmol/h/kg BW) | 2.727*** | 0.263 | 4.4347*** | 0.8642 | 0.571 | 0.613 |
| 11 | NHF-glucose | ME (MJ/kg DM) | -2.014^NS^ | 1.398 | 0.5619*** | 0.1377 | 1.046 | 0.335 |
| 13 | NHF-glucose | NHF-BHB | 3.269*** | 0.249 | 0.378* | 0.185 | 0.597 | 0.131 |

NS = not significant; * P<0.05; **P<0.01; ***P<0.001

aN α-amino-N; BW body weight; BHB β-hydroxybutyrate; C3 propionate; DM dry matter; DOM digestible organic matter; EB energy balance; ME metabolisable energy; NHF net hepatic flux; NPA net portal appearance; PDIE(N) protein digestible in the intestine as limited by the energy (nitrogen) supply; RMSE residual mean square error
